# Highly Stretchable Capacitive Sensor with Printed Carbon Black Electrodes on Barium Titanate Elastomer Composite

**DOI:** 10.3390/s19010042

**Published:** 2018-12-22

**Authors:** Eshwar Reddy Cholleti, Jonathan Stringer, Mahtab Assadian, Virginie Battmann, Chris Bowen, Kean Aw

**Affiliations:** 1 Department of Mechanical Engineering, University of Auckland, 1010 Auckland, New Zealand; echo896@aucklanduni.ac.nz (E.R.C.); j.stringer@auckland.ac.nz (J.S.); m.assadian@auckland.ac.nz (M.A.); 2 Department of Materials Engineering, Ecole Nationale Supérieure d’Ingénieurs de Caen, 14000 Caen, France; virginie.battmann@ecole.ensicaen.fr; 3 Department of Mechanical Engineering, University of Bath, BA2 7AY Bath, UK; c.r.bowen@bath.ac.uk

**Keywords:** highly stretchable sensor, printed carbon black interdigital capacitor, barium titanate

## Abstract

Wearable electronics and soft robotics are emerging fields utilizing soft and stretchable sensors for a variety of wearable applications. In this paper, the fabrication of a highly stretchable capacitive sensor with a printed carbon black/Ecoflex interdigital capacitor is presented. The highly stretchable capacitive sensor was fabricated on a substrate made from barium titanate–Ecoflex^TM^ 00-30 composite, and could withstand stretching up to 100%. The designed highly stretchable capacitive sensor was robust, and showed good repeatability and consistency when stretched and relaxed for over 1000 cycles.

## 1. Introduction

Conventional capacitive sensors are made from hard materials such as silicon, which require high processing temperatures [1,2,3] and cannot be easily integrated with wearable electronic devices and soft robotics. The ability to stretch and operate over a large strain range are critical requirements of sensors for wearable and conformal surface applications. Examples include smart skins for prostheses and health care devices for the control of activity and for monitoring the movements of the human body [4,5,6]. In addition, these sensors can also be used in the field of humanoid robotics and human–machine interactions [7,8,9,10]. The design and development of stretchable sensors can allow the measurement and quantification of physical signals generated by the human body, which would help to diagnose ailments and develop bespoke therapies for rehabilitation purposes [11,12,13,14].

Wearable sensors should ideally be flexible, stretchable, sensitive, thin, and light-weight [15]. Several methods have been used to fabricate soft sensors, such as the use of hybrid materials and structures [16], as well as nanomaterial-based electrodes, which include the use of metal and carbon nanofibers [17,18]. Moreover, electrodes with high resolution and performance are widely manufactured by printing methods [19,20]. Soft sensors are typically made from a combination of substrate and electrode parts, and silicone-based elastomers are a suitable option due to their high elongation limits and low stiffness. In addition, a substrate with a high dielectric constant is desired [21] to maximize the overall capacitance, and therefore the change in capacitance with strain. Due to the electrical conductivity requirement of the electrode that is attached to the stretchable substrate, materials of interest include thin metal films, liquid metals, and carbon-based materials, such as carbon nanotubes, graphene, and carbon black [21]. Carbon nanotubes (CNTs) have been commonly used because of their conductivity even under high strains due to the ease in creating a percolated conductive network. However, contact with a low concentration of CNTs during fabrication or damage to the sensor could lead to health risks, owing to the carcinogenic nature of CNTs [22,23]. The conductivity of strain sensor made from small flakes of graphene (GP) show pressure sensitivity. However, the sensitivity depends on the GP size [24] and the sensor also exhibits large hysteresis [25]. Carbon black (CB) has high conductivity and has shown flexibility, repeatability, and low cost [26]. The spherical morphology of CB makes it biocompatible. 

Previously fabricated capacitive sensors have used CNTs to print an interdigital capacitor (IDC) on Ecoflex substrate [21]. To circumvent the potential health risks of using CNTs—a particular concern given the potential usage of such devices in a healthcare environment—we have avoided the use of CNTs and used carbon black (CB)/Ecoflex conductive ink. Furthermore, a dispersion of 200 nm barium titanate, BaTiO_3_ (BTO) particles was used to increase the dielectric constant of the substrate and increase the sensitivity of the sensor. Other reported stretchable sensors have used silver-polymer blend electrodes [27,28], with the presented CB/Ecoflex ink showing considerably improved high-strain behavior. The choice to use an IDC structure in this work, over the parallel plate capacitor was made due to the fact that external strain and pressure affect the overlapping area and vertical distance between the parallel plates and lead to corresponding capacitance changes [21]. Moreover, the parallel plate structure limits the potential applications to rigid electronics, whereas IDC sensors extend applications to wearable electronics.

In this paper, we present the design and fabrication of a soft, flexible, lightweight, and highly stretchable capacitive sensor based on a combination of a barium titanate–Ecoflex^TM^ composite substrate and a CB/Ecoflex^TM^ electrode. The combination of stretchable substrate and the IDC allows this sensor to be highly flexible and stretchable. A CB/Ecoflex^TM^ 00-30 composite conductive ink is used to print the IDC on the BTO–Ecoflex^TM^ 00-30 composite substrate. The IDC layout has electrical contacts that can be applied to a single surface of the substrate by printing, thereby removing the need for upper and lower electrodes as required in overlapping area capacitor stretch sensors. The IDC will change in capacitance when stretched, as the spacing between the electrodes widens.

BTO was added into the Ecoflex to create a composite with increased effective dielectric constant (i.e., relative permittivity). While Ecoflex^TM^ 00-30 is flexible and stretchable, its relative permittivity is small: *ε*_r_ = 2.8 [29]. Hence, the capacitance of the printed IDC sandwiched by Ecoflex will be limited by this small capacitance value. External unwanted capacitance from associated electrical connections may lead to parasitic capacitance that would be added to the IDC capacitance, leading to large errors when translating the capacitance value to the strain. However, if the capacitance of the IDC can be significantly increased (in this case, through the addition of BTO to the Ecoflex), the external unwanted capacitance will only contribute to a smaller percentage of error, hence reducing the noise associated with the capacitance value. The BTO exhibits a high dielectric constant (*ε*_r_ ~1500–25,000, depending on the particle size and synthesis method) [30,31,32], and is dispersed uniformly in Ecoflex^TM^ 00-30 polymer matrix to increase the dielectric constant of the stretchable substrate. Here, BTO of up to 40 wt% was added into the Ecoflex to act as the highly stretchable substrate, while the stretchable IDC was made from printed CB/Ecoflex composite conductive ink.

## 2. Material Preparation and Printing Method

### 2.1. Preparation of Carbon Black/Ecoflex^TM^ 00-30 Composite Ink

In this work, CB powder with 50 nm particle size (Vulcan^®^ XC72R purchased from Fuel Cell Store, TX, USA) was selected as a conductive phase to fabricate stretchable interdigital capacitor (IDC) electrodes. Commercially available silicone-based polymer (Ecoflex^TM^ 00-30) was used as the flexible and stretchable elastomer medium. The conductivity and printability of CB/Ecoflex^TM^ 00-30 ink is dependent upon the concentration of CB in Ecoflex^TM^ 00-30, and also the amount of added thinners such as silicone oil.

The CB/Ecoflex^TM^ 00-30 ink was prepared by mixing one part of CB with five parts of Ecoflex^TM^ 00-30 by weight, and diluted with silicone oil, Besil DM 1 Plus (Wacker Chemie AG), in order to achieve the extrusion of CB/Ecoflex^TM^ 00-30 ink through the customized Lulzbot^®^ (Loveland, CO, USA) photo polymer extrusion (PPE) printer nozzle. The ink was mixed with a Kurabo planetary centrifuge mixer (Mazerustar KK-50S). The mixing ratio of CB with Ecoflex^TM^ 00-30 affects the viscosity of CB/Ecoflex^TM^ 00-30 ink. With a small ratio of CB to Ecoflex, the ink viscosity is low and easy to print, but has lower conductivity, while increasing the CB improves the conductivity but increases the ink viscosity. Here, in order to achieve repeatable printing of a high-conductivity ink, 3 mL of silicone oil was added as the thinner to 10 mL of CB/Ecoflex^TM^ 00-30 (1:5 wt%) ink.

### 2.2. Preparation of Ecoflex^TM^ 00-30 and BTO–Ecoflex^TM^ 00-30 Composite Substrates

Three types of substrates were prepared: Ecoflex^TM^ 00-30 with 0, 30, and 40 wt% BTO. The BTO nano-particles (200 nm) purchased from TPL Inc. (Albuquerque, NM, USA) were initially manually mixed with Ecoflex^TM^ 00-30, and then homogenously dispersed in the Ecoflex^TM^ 00-30 using a planetary mixer (Mazerustar KK-50S). The mixture was then poured into an acrylic mold and left to cure at room temperature for a minimum of 12 h. The loading of BTO was limited to 40 wt% based upon preliminary experiments that demonstrated that when the amount of BTO reached 50% by weight, the composite substrate took an extremely long time to cure and broke easily at the first cycle of the stretch test. A 50 wt% BTO would be approximately 14.5 vol% of the total volume, which approaches the theoretical percolation volume percentage of spheres of 16% [33]. At such high solid loadings of BTO, it is likely that there would be large agglomerated regions of BTO within the substrate, which would result in likely points of failure under mechanical loading. Fatigue testing demonstrated that substrates with 40 wt% BTO or lower in the Ecoflex survived the 1000 stretch and relax (100% strain) cycle. Hence, only 30 and 40 wt% (approximately 6.8 and 10.1 vol%) BTO in Ecoflex were examined.

### 2.3. Printing of Interdigital Electrodes

**Figure 1:**
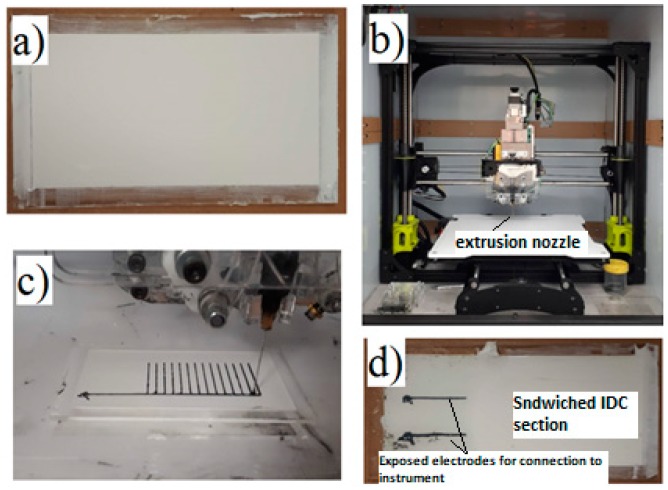
(**a**) BaTiO_3_ (BTO)–Ecoflex^TM^ 00-30 substrate, (**b**) customized Lulzbot^®^ photo polymer extrusion (PPE) 3D printer, (**c**) printing interdigital capacitor (IDC) with CB/Ecoflex^TM^ 00-30 ink, (**d**) stretchable capacitive sensor. (Note: IDC fingers spacing was 1.5 mm, 14 pairs of 20 mm long).

Figure 1 shows the steps to fabricate the sensors. The prepared BTO–Ecoflex^TM^ 00-30 composite was poured into an acrylic mold with dimensions of 75 mm × 40 mm × 1 mm and left to cure for 12 h (Figure 1a). The IDC was printed with CB/Ecoflex^TM^ 00-30 ink on the cured (0, 30, 40 wt%) BTO–Ecoflex^TM^ 00-30 composite substrate by using a customized Lulzbot^®^ photo polymer extrusion (PPE) 3D printer (Figure 1b,c), subsequently covered by a second layer of BTO–Ecoflex^TM^ 00-30 composite with corresponding amount of BTO, and cured at room temperature for a further 12 h. This led to the formation of a sealed sandwich structure for the printed IDC, leaving only sections of the two sensor IDC electrodes exposed (Figure 1d) for electrical connection.

**Figure 2:**
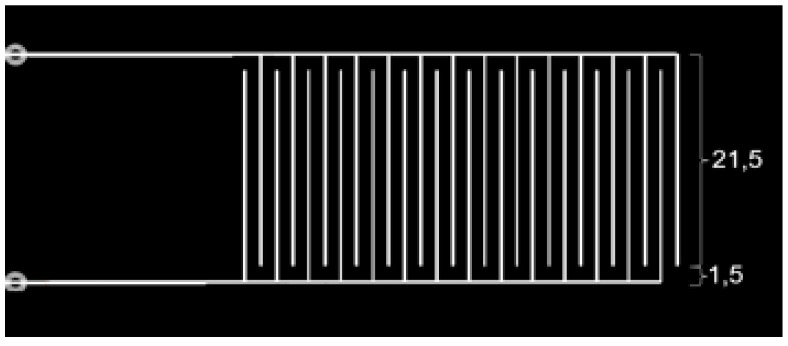
The designed dimensions of the printed IDC. Dimensions are in mm.

The designed dimensions of the printed IDC generated using a G-code simulator are shown in Figure 2. As mentioned, in this work, the IDCs were printed on three different Ecoflex substrates with different loadings (wt%) of BTO nano-particles added, as described in Section 2.2. The designed or targeted dimensions as per the G-code were: electrode spacing, *s*, of 1.5 mm, electrode width, *w*, of 0.33 mm, height, *h*, of 0.33 mm (since the extrusion needle was of gauge 23 in size) and the electrode length, *l*, of 20.0 mm.

## 3. Test Set-Up

**Figure 3:**
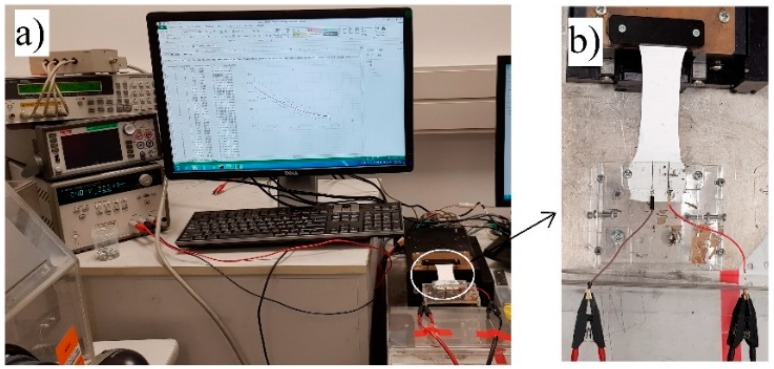
(**a**) A testing rig showing an Agilent 4263B LCR meter with a motorized stretching stage; (**b**) the sensor being stretched by the motorized stage.

The testing rig used to measure the capacitance as a function of strain is shown in Figure 3. The IDC was clamped to a motorized stage, with the visible electrode terminals on the top surface to enable connection to an Agilent 4263B LCR meter (Figure 3b). The motorized stage was designed such that one end of the sample was fixed in a stationary position, while the other end connected to the motorized stage was moved at a controlled rate to stretch the sample uniaxially. The capacitance of the IDC was measured at 10 kHz using an Agilent 4263B LCR meter, since the capacitance becomes frequency independent at ~5 kHz; this is due to a small degree of conductivity which can influence low frequency permittivity and capacitance [34]. As the capacitance of the IDC was measured as it was being stretched, a capacitance versus strain plot could be generated. The sensors were stretched up to 100% of the original length and then relaxed. The rate of both the stretching and relaxing parts of the cycle were performed at a rate of 0.7 mms^−1^, and the corresponding IDC capacitance and strain were sampled and read every 1.5 s. As this is an exploratory work, all electrical measurements were conducted on three samples for each substrate type and the average values were reported.

## 4. Results and Discussion

The performance of the sandwiched IDC as a highly stretchable capacitive sensor depends on the electrode overlap length *l*, width *w*, height *h*, and electrode spacing *s*. The actual printed dimensions of the IDC are summarized in Table 1.

**Figure 4:**
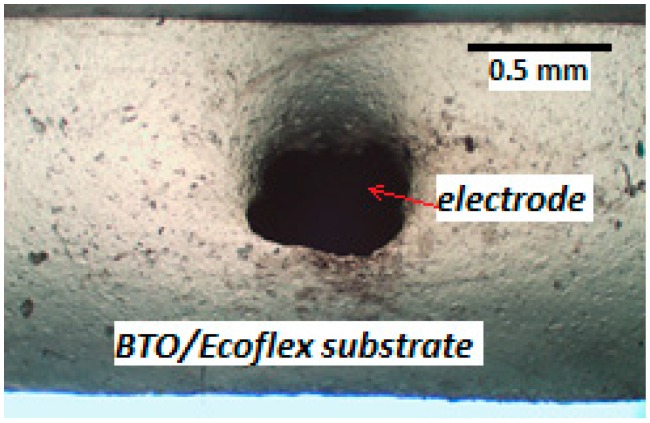
A cross section of the printed carbon black (CB)/Ecoflex electrode.

From Table 1, it can be seen that the actual dimensions of the printed IDC deviated slightly from the designed dimensions. The extruded CB/Ecoflex^TM^ 00-30 ink flattened slightly due to a combination of gravitational and capillary forces, causing the width to be wider and the height to be lower. This also made the spacing between the electrodes smaller than designed. A typical cross section of the printed electrodes is shown in Figure 4.

**Figure 5:**
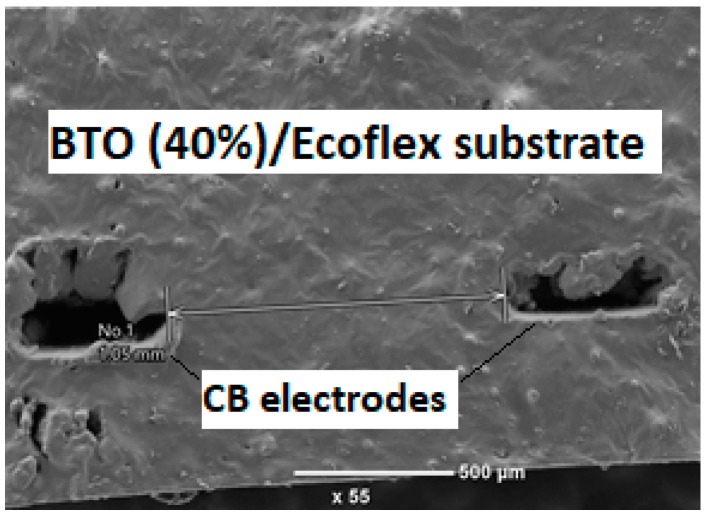
SEM image of a cross section of 200 nm BTO–Ecoflex^TM^ 00-30 composite substrate and printed CB/Ecoflex electrode.

An SEM image of a cross section of 200 nm BTO–Ecoflex^TM^ 00-30 composite substrate and printed CB/Ecoflex electrodes is shown in Figure 5. It does not show any significant surface roughness in the substrate, indicating that the BTO was reasonably well-distributed within the Ecoflex matrix.

**Figure 6:**
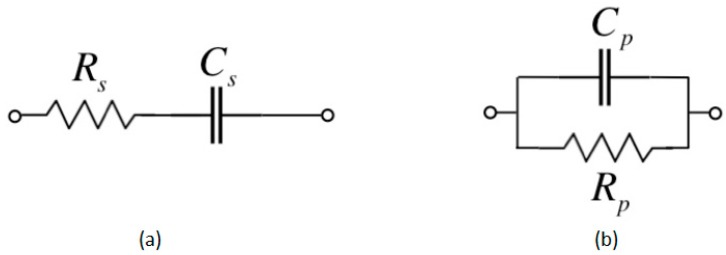
The equivalent circuit diagram of interdigital capacitive sensor (**a**) R_S_C_S_ in series (**b**) R_p_C_p_ in parallel.

An IDC can be represented with an equivalent RC circuit consisting of equivalent R_S_C_S_ in series or R_p_C_p_ in parallel as shown in Figure 6. The Agilent 4263B LCR meter can be configured to measure the equivalent R_S_, C_S_, or R_p_C_p_.

**Figure 7:**
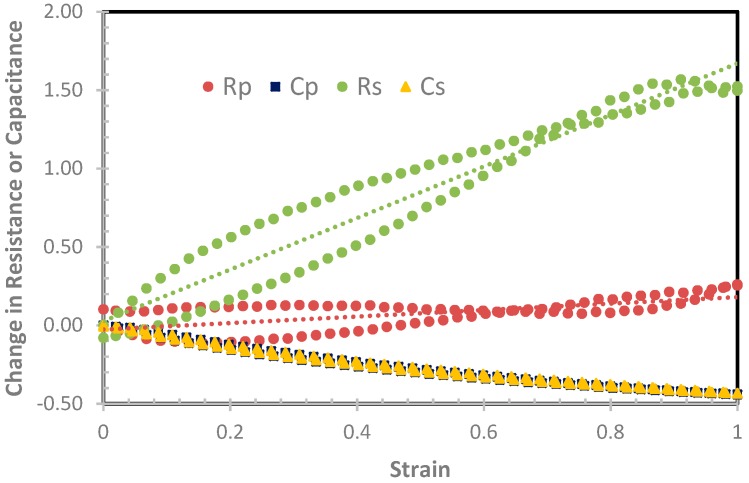
The change in R_S_, R_P_, C_S_, and C_P_ versus strain of the IDC sensors made from 40 wt% 200 nm BTO.

Figure 7 shows the plot for one sensor showing changes in R_S_, C_S_, R_P_, and C_P_ versus strain. The gauge factors based on R_S_ and R_P_ were approximately 1.7 and 0.3, respectively. The hysteresis for both R_S_ and R_P_ were extremely large. This figure shows the presence of crossovers for R_S_ and R_P_ when the sensor was stretched to 100% strain and then relaxed, and is consistent with our previous work [25]. These crossovers coupled with large hysteresis could lead to errors. Hence, R_S_ and R_P_ are not suitable parameters to represent the strain, whereas the plot of C_S_ and C_P_ versus strain did not exhibit any crossovers and had small hysteresis. However, the use of C_S_ or C_P_ did not yield a linear response, leading to a varying gauge factor from 0.4 to 0.8, which is lower than the gauge factor when R_S_ was used. There was extremely little distinction between C_S_ and C_P_, and from this point onwards only C_S_ will be reported.

The measured capacitance of the IDC sandwiched between different substrates at 0% strain is summarized in Table 2. Table 2 shows that the increase of the percentage of BTO (wt%) in Ecoflex increased the capacitance of the IDC, due to the increase of the resultant relative permittivity of the BTO–Ecoflex^TM^ 00-30 composite. 

If the relative permittivity of Ecoflex 00-30, is 2.8, the relative permittivity of the composite substrate with 30 wt% and 40 wt% BTO can be proportionally deduced to be 4.9 and 6.6 using their respective measured capacitances. However, the composite substrate relative permittivity, *ε_composite_*, could also be calculated with the Lichtenecker model, that is, $log\varepsilon_{composite}=log\varepsilon_{1}+qlog\left(\frac{\varepsilon_{2}}{\varepsilon_{1}}\right)$ [35], where *ε*_1_ and *ε*_2_ are the relative permittivity of Ecoflex and BTO (*ε*_2_ = 5000 [36]), respectively, and *q* is the volume fraction of BTO. The relative permittivities of the composite substrate calculated from the measured capacitance and the model in [35] are summarized in Table 3. This table shows that the relative permittivity of the composite calculated from measured capacitance was slightly higher than those calculated with the model in [35]. This is most likely due to the assumption of a clean two-component system in the model.

**Figure 8:**
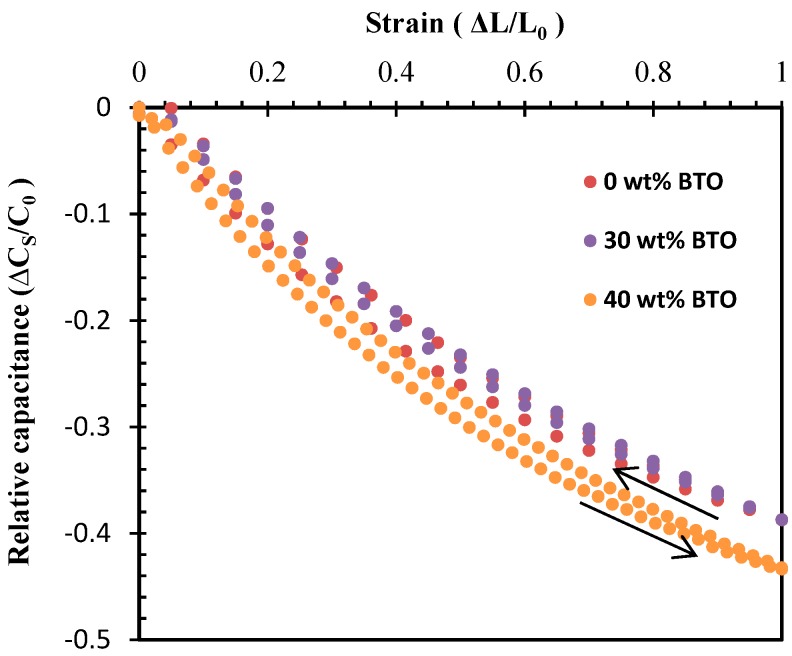
The change in capacitance (C_S_) versus strain of the IDC sensors made from 0 wt%, 30 wt%, and 40 wt% BTO.

The IDC sensor made from Ecoflex with 0 wt%, 30 wt%, and 40 wt% BTO was stretched up to 100% strain, effectively a doubling of its length, and then relaxed. The corresponding change of capacitance was determined for each stretch and then plotted in Figure 8. It is clear that the capacitance reduced non-linearly to approximately 40 wt% of the zero-strain value when stretched to 100% for sensors with 0% and 30% BTO. The sensor with 40 wt% BTO had slightly higher change in capacitance (44%) when strained to 100%. When stretched, the change in capacitance was dependent upon the spacing, *s*, between the electrodes and the overlapping length, *l*, of the IDC. This is likely due to the non-linear stress–strain behavior of the elastomeric matrix. Further, the hysteresis loop was relatively small for all substrate types, with the 40 wt% BTO substrate showing the largest hysteresis (~4%) among the three substrates.

The Poisson’s ratios of the substrates when strained are summarized in Table 4. The change in capacitance for a unit strain will be higher for a substrate with a higher Poisson’s ratio, as capacitance reduces with the IDC overlapping length, *l*. The addition of BTO led to a reduction in Poisson’s ratio, but this was countered by increasing the relative permittivity of the composite substrate and was shown with the slightly higher change in capacitance in the sensor with 40 wt% BTO despite having a lower Poisson’s ratio.

**Figure 9:**
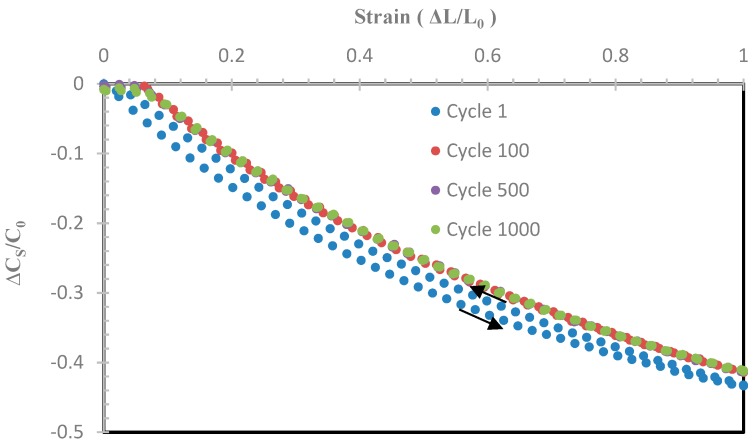
The relative change in capacitance strained up to 100% after 1, 100, 500 and 1000 stretch/relax cycles.

The repeatability and reliability of this highly stretchable IDC sensor with the substrate made from 40 wt% BTO in Ecoflex was evaluated by stretching up to 100% and then relaxing to 0% for 1000 cycles with simultaneous capacitance measurement. Representative plots of relative capacitance change (ΔC_s_/C_0_) versus strain at stretching cycle 1, 100, 500, and 1000 are shown in Figure 9. Note that C_0_ is the initial resistance at 0% strain.

**Figure 10:**
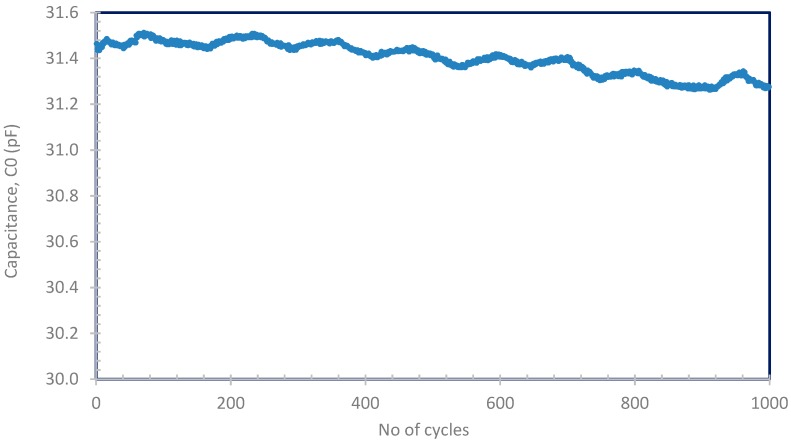
The zero-strain capacitance of the stretch sensor with 40 wt% BTO with respect to stretch/relax cycles.

From Figure 9, it is clear the sensor performance was repeatable over 1000 cycles. In this plot only Cycle 1 showed hysteresis and this then reduced significantly as the stretch/relax cycle progressed. This indicates that the composite was stable at high strain, since changes in the distribution of the high-permittivity BTO filler particles are likely to lead to changes in the capacitance of the device. The capacitance of the sensor with 40 wt% BTO at zero-strain, C_0_, for each stretch/relax cycle were plotted up to 1000 cycles as shown in Figure 10. From this plot, C_0_ remained between 31.4 ± 0.1 pF. This demonstrated that this stretch sensor remained mechanically robust and did not deform significantly after 1000 cycles, as C_0_ remained relatively constant.

**Figure 11:**
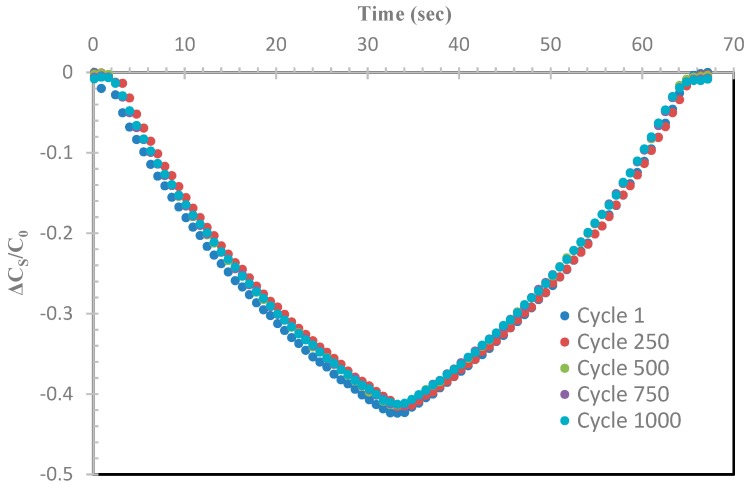
The ΔC_S_/C_0_ versus time as the sensor was stretched to a maximum of 100% strain.

Figure 11 shows a snapshot of ΔC_S_/C_0_ versus time for selective cycles for the sensor made from substrate with 40 wt% BTO–Ecoflex. The consistency of the ΔC_S_/C_0_ over stretch/relax cycles demonstrates the stability and reliability of the sensor.

**Figure 12:**
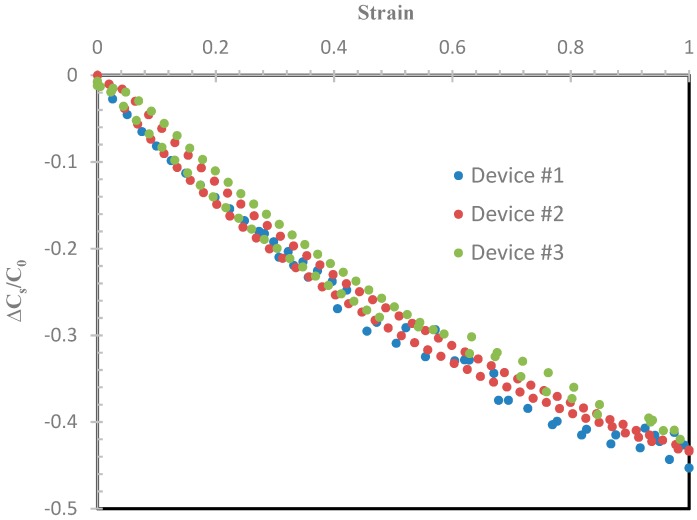
The worst-case ΔC_S_/C_0_ versus strain plots of the three sensors stretched to 100% and relaxed.

To illustrate the spread of the data over three samples, Figure 12 provides a snapshot of the worst-case plots of the ΔC_S_/C_0_ for the sensor with 40 wt% BTO being stretched up to 100% strain and then relaxed. This worst-case result shows that the variation between the three different sensors was minimal, and was calculated to be within 5%.

The work presented here demonstrated better performance compared to recently published work [21,37]. In [21], polydimethylsiloxane (PDMS, Sylgard 184) was used, but was limited to a strain of 50%. This was most likely due to the failure of Sylgard 184 at high strain when compared to the Ecoflex silicone elastomer used in this work. A capacitance change of 36% at 50% strain was reported, although there was no report on the hysteresis and reliability of the sensor. In [37], silver particles in Ecoflex 00-50 were used, and claimed strains of up to 100% (with no change in capacitance reported at this strain), but only exhibited a 16% change in capacitance at 63% strain, in comparison to our sensor with capacitance change of ~32% at the same 63% strain, demonstrating the increased sensitivity of the sensor fabricated in this work. Further, the capacitance at 0% strain started to increase after 20 stretch/relax cycles, indicating poor stability.

## 5. Conclusions

Our work on the use of a printed CB/Ecoflex^TM^ 00-30 interdigital electrode sandwiched between a BTO–Ecoflex^TM^ 00-30 stretchable substrate as a large-strain sensor based on change in capacitance was presented in this paper. An increase in absolute capacitance by adding the 200 nm BTO with Ecoflex^TM^ 00-30 was clearly observed. The advantages of having higher absolute capacitance are the ease on the demand upon the electronics to measure the capacitance and less susceptibility to external unwanted capacitance. The gauge factor based on the measured capacitance, C_S_ or C_P_ ranged from 0.4 to 0.8 due to the non-linear response. The response of the sensor based on the equivalent resistance, R_S_ or R_P_ yielded a linear response with higher gauge factor, but the presence of crossover and higher hysteresis would prove challenging in real-life applications. These large-strain sensors exhibited high repeatability and consistency when repeatedly stretched up to 100% strain and relaxed over 1000 cycles with the addition of BTO into Ecoflex of up to 40 wt %. The key points are that the reproducibility and long-term stability of the samples with time and cycles, which are critical criteria, and these are examined in Figure 8, Figure 9, Figure 10 and Figure 11 to demonstrate the performance of the material.

## Figures and Tables

**Table 1:** The actual dimensions of the printed IDC, dimensions in mm.

Dimension (mm)	Overlap Length, *l*	Width, *w*	Height, *h*	Spacing, *s*
Average	19.50 ± 0.06	0.58 ± 0.13	0.26 ± 0.07	1.06 ± 0.01
Designed dimension	20.00	0.33	0.33	1.50

**Table 2:** The measured capacitance of the IDC sandwiched between different substrates.

Sandwiching Substrate	Capacitance of IDC (pF)
0 wt.% BTO (0 vol %)	13.27
30 wt.% BTO (6.8 vol %)	23.35
40 wt.% BTO (10.1 vol%)	31.40

**Table 3:** Comparison of the relative permittivity of the composite substrate.

Sandwiching Substrate	Calculated from Measured Capacitance, *ε_composite_*	Calculated from Formula in [35]
0 wt.% BTO (0 vol%)	2.8	2.8
30 wt.% BTO (6.8 vol%)	4.9	4.6
40 wt.% BTO (10.1 vol%)	6.6	5.9

**Table 4:** Poisson’s ratio of the substrate when strained.

Sandwiching Substrate	Poisson Ratio
0 wt.% BTO (0 vol %)	0.38
30 wt.% BTO (6.8 vol %)	0.31
40 wt.% BTO (10.1 vol%)	0.29
